# Using *in vitro* gastrointestinal (GI) tract digestive products to investigate fermentation dynamics of *Lactobacillus rhamnosus* GG, *Bifidobacterium animalis* subsp. *lactis* BB-12, and *Escherichia coli*

**DOI:** 10.1080/29933935.2026.2620183

**Published:** 2026-01-26

**Authors:** He Lyu, Saartje Hernalsteens, Qijun Zheng, Aibing Yu, Xiao Dong Chen

**Affiliations:** a Life Quality Engineering Interest Group, School of Chemical and Environmental Engineering, College of Chemistry, Chemical Engineering and Material Science, Soochow University, Suzhou, People's Republic of China; b Department of Chemical Engineering and Biological Engineering, Monash University, Clayton, Victoria, Australia; c Pro-health Instrumentation (Suzhou) Co., Ltd., Suzhou, People's Republic of China; d Centre for Smart Process Engineering, Great Bay University, Dongguan, People's Republic of China; e Institute of Biopharmaceutical and Health Engineering, International School of Postgraduates, Tsinghua University, Shenzhen, People's Republic of China

**Keywords:** Human milk, infant formula, digestive products, *in vitro* colonic fermentation, Soft elastic tubular reactor, *Lactobacillus rhamnosus* GG, *Bifidobacterium animalis* subsp. *lactis* BB-12, *Escherichia coli*

## Abstract

Gastrointestinal digestion shapes nutrient availability and microbial substrates in early life, yet how milk digestion products regulate probiotic–pathogen dynamics remains unclear. Using *in vitro* digestion products of human milk (HM), infant formula (IF), and control milk (CM), we evaluated the fermentation responses of *Lactobacillus rhamnosus* GG (LGG), *Bifidobacterium animalis* subsp. *lactis* BB-12 (BB-12), and *Escherichia coli* (*E. coli*) in static reactors (ST) and peristalsis-mimicking soft elastic tubular reactors (SETR). HM-derived residues selectively enhanced probiotic activity by increasing reducing-sugar utilization, acidification (ΔpH), and overall metabolic output by 110% (LGG) and 130% (BB-12), while suppressing *E. coli* growth by 28%. In the absence of digestive products, LGG and BB-12 declined by 0.99 and 3.19 log units, whereas *E. coli* increased by 0.33 log. Mechanical compression in SETR further boosted BB-12 metabolic output by 23% compared with ST. These results demonstrate that infant milk digestion products differentially regulate microbial strategies. We acknowledge that this simplified three-strain model does not capture full gut community complexity, and broader consortia studies are needed for future validation.

## GRAPHICAL ABSTRACT

**Figure:**
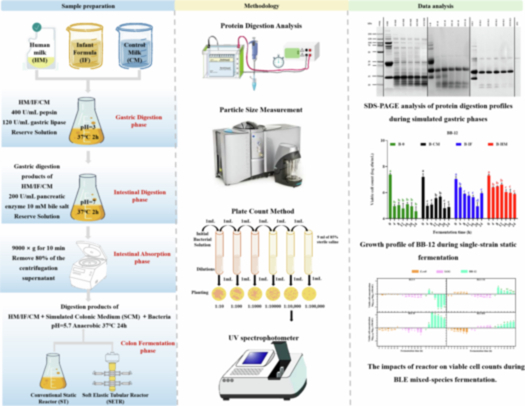

## Introduction

Recent advances in gut microbiota research have revealed its critical role in maintaining host homeostasis and modulating disease progression. As a highly dynamic ecosystem, the gut microbiota forms a symbiotic network with the host through metabolic interactions, immune modulation, and neuroendocrine communication.^1-3^ Clinical and experimental studies have demonstrated that gut microbial imbalance contributes to various host disorders, underscoring the importance of diet as a key modulator of microbial ecology. Consequently, nutritional composition has become a focal point in understanding how early-life feeding shapes microbial succession and intestinal health.

In the field of infant nutrition, probiotics have gained significant attention due to their role in modulating gut barrier function and immune responses. *Lactobacillus rhamnosus* GG (LGG) and *Bifidobacterium animalis* subsp. *lactis* BB-12 (BB-12) are clinically validated probiotic strains,^4^^,^^5^ known for their ability to enhance short-chain fatty acid production and inhibit pathogen colonization in adult studies. However, their metabolic interactions with complex food matrices within the infant gastrointestinal environment remain poorly characterized. This knowledge gap is particularly significant given the developmental differences between infant and adult gut microbiota. Concurrently, *Escherichia coli* (*E. coli*) exhibits a dual role in gut homeostasis, commensal strains contribute to vitamin synthesis and colonization resistance, whereas pathogenic strains pose a threat to infant gut health.^6^ The strain used in this study, BNCC 133264, is a non-pathogenic commensal *E. coli* isolate widely applied in gut modeling systems to represent early-life facultative anaerobes. This functional antagonism between probiotics and pathogens provides a unique perspective for investigating the regulatory effects of dietary residues on microbial balance in early life.

Food matrices serve as key drivers of gut microbiota functional differentiation. Human milk (HM), the optimal nutritional source for infants, contains bioactive components including human milk oligosaccharides and immunoglobulins that selectively promote *Bifidobacterium* growth while inhibiting pathogenic colonization.^7^^,^^8^ Infant formula (IF) attempts to mimic HM’s functional effects through the incorporation of prebiotics and other bioactive ingredients. However, processing-induced alterations in protein and lipid structures may modify digestive byproducts, thereby influencing microbial interactions.^9^^,^^10^ These compositional and functional disparities highlight the need to compare HM and IF digestion residues in shaping infant gut microbiota, particularly during early intestinal development.

Previous *in vitro* and *in vivo* studies have extensively compared the effects of HM and IF on infant gut microbial composition. These studies consistently report that HM feeding enriches *Bifidobacterium* and *Lactobacillus* populations, whereas IF feeding often increases microbial diversity and the relative abundance of facultative anaerobes such as *Enterobacteriaceae*.^11-13^ Nevertheless, most of these investigations rely on fecal analyzes or simplified substrate fermentations, which provide limited mechanistic insight into how digestive transformation of milk components modulates microbial metabolism. In contrast, *in vitro* infant gastrointestinal fermentation models offer a controllable platform for studying food–microbe interactions under defined conditions. Yet, existing models often employ isolated nutrients rather than complex digestion residues and are largely based on adult digestive parameters.^14^^,^^15^ Importantly, *in vitro* systems cannot replicate key host-mediated processes—including epithelial barrier responses, mucosal immune signaling, and systemic physiological effects—thus limiting their ability to fully recapitulate in vivo infant gut environments.

To address these limitations, the present study builds upon previous *in vitro* infant fermentation models by incorporating infant-specific digestive parameters and employing a soft elastic tubular reactor (SETR) capable of mimicking colonic peristalsis. This dynamic system enhances the physiological relevance of the *in vitro* setup by reproducing mechanical shear and nutrient flow—two critical factors influencing microbial colonization and metabolite exchange. The integration of SETR with infant gastrointestinal digestion products provides a novel platform to explore how milk-derived substrates shape microbial fermentation and metabolic outcomes.

To ensure mechanistic clarity and reproducibility, this study employed a reductionist approach by selecting three representative bacterial strains instead of complex fecal microbiota: *Lactobacillus rhamnosus* GG (LGG) and *Bifidobacterium animalis* subsp. *lactis* BB-12 (BB-12) as well-characterized probiotics, and a commensal *Escherichia coli* strain (BNCC 133264) as a model facultative anaerobe with both commensal and pathogenic potential. This simplified design enables precise assessment of substrate-specific microbial responses while minimizing confounding effects from undefined microbial communities. Although reduced in complexity, such a targeted system provides fundamental mechanistic insights that can later inform more comprehensive community-level investigations. Accordingly, the present study investigated the proliferation and metabolic behavior of LGG, BB-12, and *E. coli* during *in vitro* simulated colonic fermentation using gastrointestinal digestion products of human milk (HM), infant formula (IF), and control milk (CM). The objective was to elucidate how milk-derived digestive residues differentially regulate probiotic and pathogenic bacterial growth and metabolism, and to determine whether the SETR system provides a more physiologically relevant representation of infant gut fermentation dynamics compared with conventional static (ST) systems. Collectively, this research bridges the gap between nutritional digestion and microbial fermentation in early life. By integrating milk-specific digestion profiles with a dynamic infant fermentation platform, it advances current understanding of how early dietary exposures modulate gut microbial ecology and contributes to the development of improved nutritional strategies for infant health.

## Material and methods

### Sample preparation

HM samples were collected under ethical approval from Soochow University (SUDA20240129H01) and stored at −40 °C until use. HM was obtained from three healthy donors at 2–4 months postpartum, corresponding to the mature lactation stage. Infant formula (IF, Aptamil Profutura, Netherlands) was reconstituted as a 15% (w/w) aqueous solution according to the manufacturer’s instructions. The detailed nutritional composition of the IF used in this study is provided in Supplementary Table S1. Control milk (CM) was formulated in-laboratory to approximate a simplified milk matrix, containing 60 g/L lactose, 10 g/L casein, 20 g/L whey protein, and 1 g/L mucin. All samples were equilibrated at 37 °C for 30 min and adjusted to pH 3.0 prior to digestion.

### Reagent preparation

Simulated gastric fluid (SGF) and intestinal fluid (SIF) were prepared as described by Minekus et al.^14^ with infant-specific modifications. SGF (pH 3.0) contained 400 U/mL pepsin (P7125, Sigma, USA) and 120 U/mL gastric lipase (80612, Sigma, USA) to reflect infant digestive physiology.^16^ SIF (pH 7.0) included 20 U/mL pancreatin (8 × USP, Sigma, USA) and 5 mM bile salts (Solarbio Bile Salts No. 3, China), accounting for reduced infant bile secretion. Fluids were stored at 4 °C and pre-warmed to 37 °C before use. The chosen enzyme activities and bile salt concentration were based on validated infant gastrointestinal physiology data and published *in vitro* infant digestion protocols,^17^ ensuring that digestion products are physiologically relevant for downstream microbial fermentation. Simulated colonic medium (SCM)^18^ contained (per liter): 2.0 g peptone, 4.0 g yeast extract, 0.1 g NaCl, 40.0 mg K₂HPO₄, 40.0 mg KH₂PO₄, 10.0 mg MgSO₄, 10.0 mg CaCl₂, 2.0 g NaHCO₃, 0.46 g cysteine-HCl, 0.5 g bile salts, 2.0 mL Tween 80, and 10.0 μL vitamin K₁ (pH 5.7).

### Strain activation

*Lactobacillus rhamnosus* GG (LGG; Culturelle®, USA), *Bifidobacterium animalis* subsp. *lactis* BB-12 (Chr. Hansen®, Denmark), and *Escherichia coli* (BNCC 133264, China) were selected to model probiotic-pathogen interactions in infant gut ecosystems.^19-21^ BNCC 133264 is a non-pathogenic commensal strain commonly used in infant gut modeling to represent facultative anaerobic colonizers. LGG and BB-12 were cultured anaerobically in MRS broth (BB-12 with 0.05% cysteine-HCl), while *E. coli* was grown aerobically in Luria Bertani (LB) medium. Growth curves were derived from Log CFU/OD600 measurements. After 24 h (LGG, BB-12) or 8 h (*E. coli*), cultures were standardized to 4.7 × 10⁸ CFU/mL, a concentration chosen to match early-life facultative anaerobe densities and to ensure uniform starting biomass across strains for valid comparisons.

### Fermentation design

Single-strain (LGG, BB-12, *E. coli*) and mixed-species fermentations (BB-12- or *E. coli*-dominant) were conducted in SCM supplemented with HM, IF, or CM digesta. Anaerobic conditions (37 °C, 24 h) were maintained throughout.

### *In Vitro* gastrointestinal digestion and absorption

Simulated gastric digestion was performed by mixing CM, IF or HM with simulated gastric fluid (SGF) in a 1:1 (v/v) ratio. The pH was adjusted to 3.0 using 6 mol/L HCl, and maintained within ± 0.1 by readjustment every 30 minutes throughout the 2-hour incubation. Samples were incubated at 37 °C with continuous agitation at 100 rpm to mimic infant gastric motility. To capture time-dependent changes during digestion, samples were collected at 0, 30, 60, 90, and 120 minutes, which are denoted as G-0, G30, G60, G90, and G120, respectively. Following gastric digestion, the chyme was neutralized to pH 7.0 using 1 mol/L NaOH in preparation for intestinal digestion. For the intestinal phase, the gastric chyme was combined with simulated intestinal fluid (SIF) in a 1:1 (v/v) ratio and incubated under the same conditions (37 °C, 100 rpm) for 2 hours, with the pH stabilized at 7.0 through periodic adjustment every 30 minutes. Time-specific intestinal digestion samples were collected at 0, 30, 60, 90, and 120 minutes, denoted as I0, I30, I60, I90, and I120, respectively. All digestive fluids and enzyme concentrations were prepared following the infant-specific modifications described in the Reagent Preparation section, which were designed to replicate key physiological features of infant digestion, including reduced gastric acidity, lower bile salt concentrations, and age-adjusted enzyme activities. At the end of intestinal digestion, samples were centrifuged at 9000 × g for 10 minutes, and 20% of the digest, including both supernatant and pellet, was retained for subsequent colonic fermentation.

### *In vitro* colonic fermentation

Activated bacterial cultures (*E. coli*, LGG, BB-12) were inoculated into simulated colonic medium (SCM) at an initial concentration of 4.0 × 10⁷ CFU/mL. Digestion products derived from HM, IF, or CM were added individually to the fermentation systems, while SCM without digestion products served as the blank control. All fermentations were conducted anaerobically at 37 °C for 24 hours, with biological triplicates performed for each condition. Two reactor designs were compared in this study: a conventional static reactor (ST) and a soft elastic tubular reactor (SETR) developed by Pro-health Instrumentation (Suzhou) Co., Ltd. (Suzhou, China).

**Figure 1.**
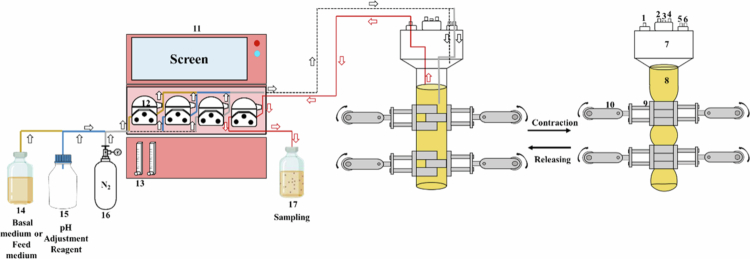
Schematic diagram of the Soft Elastic Tubular Reactor (SETR) in this study. (1) Sampling port; (2) Temperature probe; (3) Dissolved oxygen (DO) probe; (4) pH probe; (5) Feed port; (6) Gas inlet; (7) Rigid reactor vessel; (8) Soft silicone reactor model; (9) Compression plate assembly; (10) Electromechanical drive unit; (11) Fermentation control system; (12) Peristaltic pump; (13) Gas regulation valve; (14) Feed medium bottle; (15) pH adjustment reagent bottle; (16) Nitrogen gas cylinder; (17) Sample collection bottle.

The soft elastic tubular reactor (SETR) was designed as a biomimetic fermentation system inspired by the concentric muscular contractions of the human intestine, where peristaltic movements promote efficient mixing and propulsion of luminal contents. This concept was realized through cyclic deformation of flexible silicone tubing, in which periodic compression of the tube walls reproduced the dynamic mechanical environment of the infant colon (Figure 1). The SETR consisted of a soft silicone tubular chamber enclosed within a rigid support vessel. Two synchronized pusher plates, positioned 7 cm apart, moved centripetally at a frequency of six compressions per minute, thereby inducing rhythmic squeezing and relaxation of the tubing. This operating frequency was selected based on hydrodynamic analyzes, which demonstrated that such deformation patterns optimize internal fluid circulation and ensure rapid homogenization of suspended microbial and nutrient components.^22^ To maintain physiological relevance, the SETR was equipped with temperature, pH, and dissolved oxygen (DO) probes connected to an automated fermentation control unit. Anaerobic conditions were achieved by continuous nitrogen purging through a regulated gas inlet, while peristaltic pumps supplied digestive products and pH adjustment reagents via designated feed ports. Samples were collected aseptically from a sealed sampling port at predetermined time points.

## Nomenclature

Fermentation systems were labeled as follows:

Single-strain fermentation process: Strain initials + digestion products type (e.g., *E. coli* single-strain fermentation involving HM: E-HM).

Mixed-species fermentation uses the initials of all involved strains, with the dominant strain listed first + digestion products type (e.g., *E. coli:* LGG: BB-12 = 10:1:1 mixed-species fermentation involving HM: ELB-HM).

### Analytical methods

a. **Protein Digestion Analysis**

Gastric and intestinal digesta (HM, IF, CM) collected at 0, 30, 60, 90, and 120 min were analyzed by sodium dodecyl sulfate-polyacrylamide gel electrophoresis (SDS-PAGE).^23^ Protein concentrations were normalized to 4 mg/mL with deionized water. Samples were combined with NuPAGE™ LDS Sample Buffer (4×) and NuPAGE™ Sample Reducer (10×) in a 13:5:2 (v/v/v) ratio, heated at 70 °C for 10 min, and loaded (10 µL per lane) onto 12% Bis-Tris gels. A pre-stained protein ladder (5 µL, 10–250 kDa) was included for molecular weight calibration. Electrophoresis was conducted at 200 V for 50 min using Tris-MES-SDS running buffer. Gels were stained with 0.1% (w/v) Coomassie Brilliant Blue R-250 in 10% (v/v) acetic acid and 40% (v/v) ethanol, followed by destaining in 10% ethanol and 7.5% acetic acid.

b. **Particle Size Measurement**

The average particle size of digesta was determined using a laser diffraction particle analyzer (Mastersizer 3000, Malvern Instruments Ltd, UK). Measurements were performed in quintuplicate.

c. **Viable Cell Count**

The viable cell count (VCC) was determined using the plate count method. Samples were diluted with sterile normal saline (0.85%, w/v) through serial dilutions (10^1^ to 10^6^). From each dilution, 100 μL was transferred to 900 μL of sterile normal saline, and 10 μL of the suspension was spotted onto agar plates. The plates were incubated at 37 °C without agitation, and experiments were conducted in triplicate. The number of viable bacterial cells in each sample was counted and reported as log CFU/mL.

d. **pH and Reducing Sugar Quantification**

pH values were measured using a calibrated benchtop pH meter (MP511, Shanghai, China). Reducing sugar content was determined via the 3,5-dinitrosalicylic acid (DNS) method.^24^ Supernatants (300 µL), obtained by centrifuging fermentation samples at 8000 × g for 10 min, were mixed with 600 µL DNS reagent (1% 3,5-dinitrosalicylic acid, 0.2% phenol, 0.05% Na2SO3 and 1% NaOH aqueous solution). The mixture was heated at 100 °C for 5 minutes and subsequently mixed with 3.6 mL of distilled water. After cooling to room temperature, the absorbance was measured at 540 nm using a UV spectrophotometer (MAPADA, UV-1800B, Shanghai, China).

A glucose standard curve was used for quantification, consistent with standard DNS protocol. Because the digestion of HM, IF, and CM yielded different initial reducing sugar levels, all reducing sugar concentrations were normalized to their respective t = 0 values to evaluate fermentation-associated utilization rates. This normalization approach accounts for matrix-specific differences and enables meaningful comparison of reducing sugar dynamics within each milk group.

e. **Amino Acid and Free Fatty Acid Assays**

Total amino acids were quantified using the o-phthalaldehyde (OPA) method.^25^ Samples (400 µL) were precipitated with 15% (w/v) trichloroacetic acid (400 µL), centrifuged at 15,000 × *g* for 10 min, and mixed with OPA reagent (3 mL). Absorbance was measured at 340 nm after 2 min incubation. Free fatty acids (FFAs) were analyzed using a commercial non-esterified fatty acid (NEFA) assay kit (Nanjing Jiancheng Bioengineering Institute, China), following manufacturer protocols.

### Statistical analysis

All experiments were conducted in biological triplicate unless otherwise stated. For each measured variable (microbial activity, pH, reducing sugars, amino acids, and free fatty acids), normality of residuals was evaluated separately for each treatment group (0, CM, IF, HM) using the Shapiro–Wilk test. Groups that met normality assumptions (*p* > 0.05) were analyzed using repeated-measures ANOVA, followed by Bonferroni correction for pairwise time-point comparisons. Groups that did not meet normality assumptions (*p* < 0.05) were analyzed using the Friedman non-parametric repeated-measures test, followed by Benjamini–Hochberg false discovery rate (FDR) correction. All statistical analyzes were performed using SPSS 23 and Python (SciPy). Statistical significance was defined as *p* < 0.05 after correction. A full summary of normality results and the corresponding statistical methods for each dataset is provided in Supplementary Table S2.

## Results

### Different food characteristics of digestion processes

**Figure 2.**
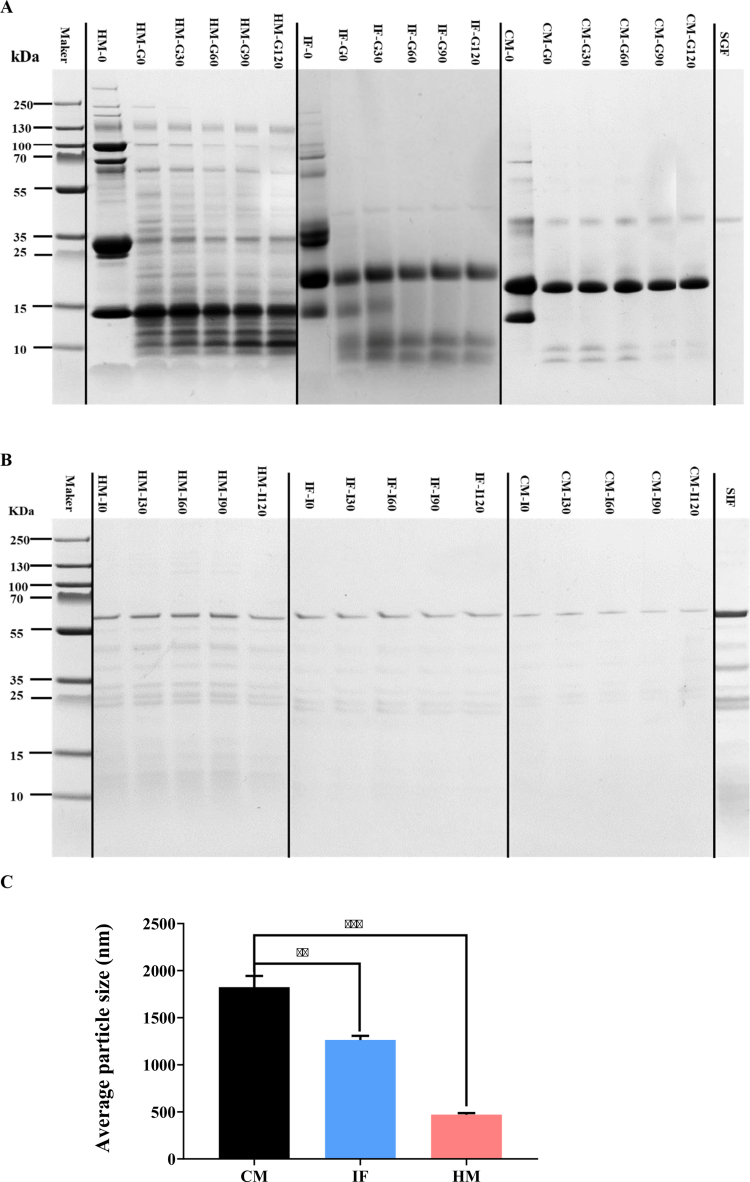
SDS-PAGE analysis of protein digestion profiles during simulated gastrointestinal phases. (A) Gastric phase (pH 3.0, 2 h): Lanes 2, 8, and 14 show native protein profiles of undigested human milk (HM), infant formula (IF), and control milk (CM). Lanes 3-7 display HM hydrolysates at t = 0, 30, 60, 90, and 120 min; lanes 9-13 show IF hydrolysates at identical time points; lanes 15-19 present CM hydrolysates. Lane 20 contains simulated gastric fluid (SGF) control. (B) Intestinal phase (pH 7.0, 2 h): Lanes 2-6 contain HM hydrolysates at t = 0, 30, 60, 90, and 120 min; lanes 7-11 show IF hydrolysates; lanes 12-16 display CM hydrolysates. Lane 17 shows simulated intestinal fluid (SIF) control. Dashed lines indicate splice boundaries for cross-sample comparison of digestibility differences. (C) Mean particle size distribution of digestion products after simulated gastrointestinal phases treatment: HM (red), IF (blue), CM (black). Values represent mean ± SD (*n* = 3 biological replicates). Statistical significance: *p < 0.05, **p < 0.01, ***p < 0.001.

SDS-PAGE profiling demonstrated distinct proteolytic dynamics across human milk (HM), infant formula (IF), and control milk (CM) during simulated gastrointestinal digestion. HM exhibited a broad molecular weight (MW) distribution (15–250 kDa), with prominent bands corresponding to *α*-lactalbumin (15 kDa), *β*-lactoglobulin (25–35 kDa), lactoferrin/serum albumin (50–70 kDa), immunoglobulin aggregates (100 kDa), and milk fat globule membrane (MFGM) proteins (130–250 kDa). Gastric digestion induced rapid degradation of high-MW proteins (>100 kDa) in HM, with partial retention of *β*-lactoglobulin and generation of low-MW peptides (<20 kDa), while IF and CM proteins were fully hydrolyzed to sub-20 kDa fragments (Figure 2A). During intestinal digestion, HM retained partially degraded medium-MW peptides (25–50 kDa), whereas IF and CM showed near-complete proteolysis to trace low-MW residues (Figure 2B). Particle size analysis aligned with these patterns. HM formed the smallest colloidal aggregates (470.15 ± 11.65 nm), reflecting efficient proteolysis, whereas CM exhibited the largest micelles (1825.00 ± 84.00 nm, *p* < 0.001 vs. HM), indicative of casein-dominant structures (Figure 2C).

### Single-strain static fermentation responses to simulated GI digestion products

**Figure 3.**
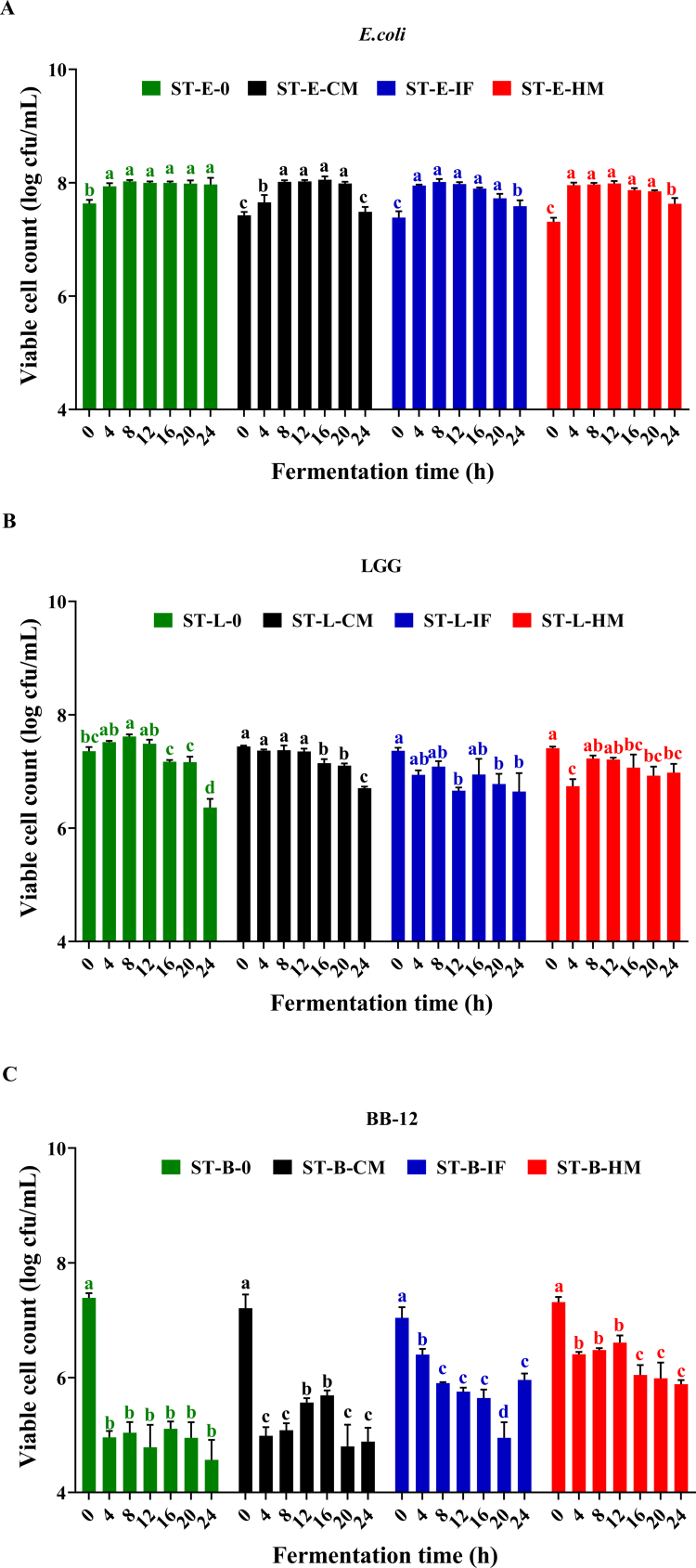
Growth profile of (A) *E. coli* in ST; (B) LGG in ST; (C) BB-12 during single-strain static fermentation. Each time point is an average of 3 replicates and the errors bars indicate standard deviation. Red represents HM, blue represents IF, black represents CM, and green represents the control without digestion products. Different lowercase letters represent significant differences between samples at different fermentation times within the same fermentation mode (*p* < 0.05).

*In vitro* static reactor (ST) fermentation assays revealed distinct growth dynamics among *E. coli*, LGG, and BB-12 in response to digested residues from control milk (CM), infant formula (IF), and human milk (HM) (Figure 3). For *E. coli* (Figure 3A), bacterial viability surged within the initial 4 h across all groups, followed by stabilization with minimal intergroup differences until 16 h. Notably, post-20 h declines occurred in residue-supplemented groups (E-CM: −0.57 log, E-IF: −0.31 log, E-HM: −0.08 log CFU/mL at 24 h vs. 16 h; *p* < 0.05), suggesting late-phase inhibitory effects of digestive byproducts. LGG (Figure 3B) exhibited substrate-dependent adaptation: while the no-residue control (L-0) maintained viability for 12 h before sharp decline (−0.99 log at 24 h vs. 0 h; *p* < 0.001), HM residues (L-HM) induced transient metabolic reprogramming marked by an initial viability drop (−0.67 log at 4 h; *p* < 0.001) followed by recovery, achieving the highest endpoint viability among groups. BB-12 (Figure 3C) demonstrated limited substrate utilization capacity, with rapid decline in CM and no-residue groups post-4 h. IF and HM residues transiently supported higher viability (peak at 4 h), though HM sustained superior persistence, indicating strain-specific metabolic flexibility. Collectively, digestive residues differentially modulated microbial survival strategies—exerting late-phase suppression on *E. coli*, inducing biphasic adaptation in LGG, and sustaining conditional persistence in BB-12, with HM-derived components showing optimal probiotic maintenance.

### Mixed-species static fermentation responses to simulated GI digestion products

**Figure 4.**
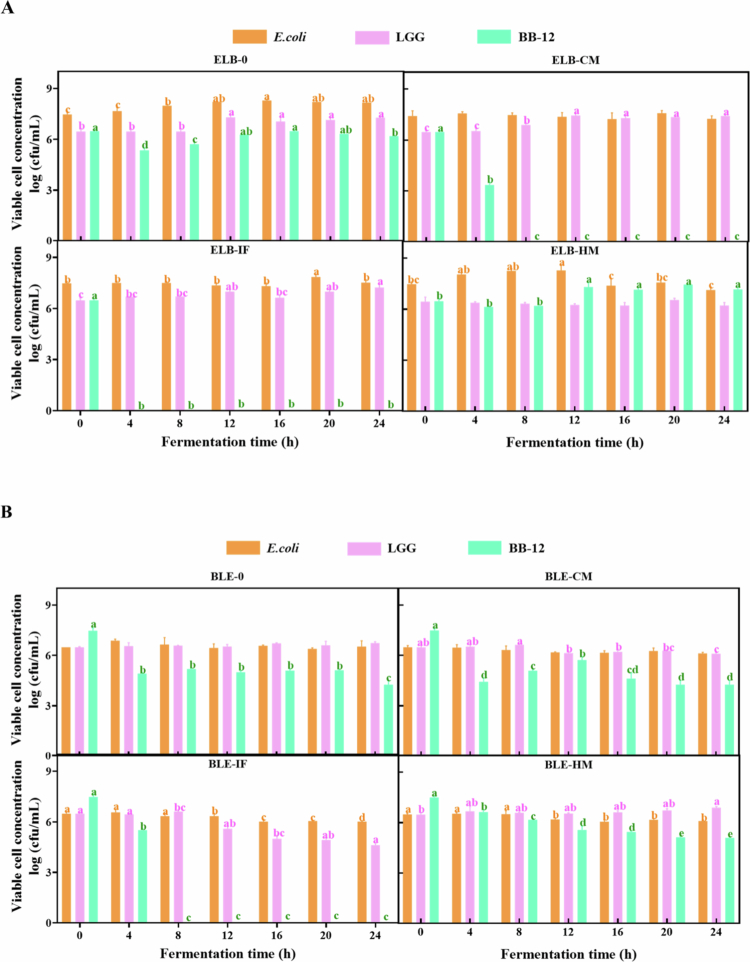
Effect of different digestion products on the viable cell count (log CFU) of each strain during mixed-species fermentation with a strain ratio of *E. coli*: *LGG*: BB-12 (ELB) = 10:1:1 (A). Strain ratio BB-12: LGG: *E. coli* (BLE) = 10:1:1 (B). Yellow represents *E. coli*, pink represents LGG, and green represents BB-12. Different uppercase letters represent significant differences between samples at different fermentation times within the same fermentation mode (*p* < 0.05).

Single-strain fermentation serves as a comparative benchmark for mixed-species fermentation, enabling a deeper understanding of the specific roles and contributions of each bacterial strain during the process. In mixed-species fermentation dominated by *E. coli* (Figure 4A), the viable cell counts of *E. coli* and LGG increased by 0.7 and 0.82 log N/N0 (*p* < 0.05), respectively, in the absence of digested products (ELB-0), whereas BB-12 exhibited a decline of 0.29 log N/N0 (*p* < 0.05) (Table 1). The growth patterns of *E. coli* and LGG were similar, characterized by an initial stabilization phase, followed by a significant increase, and subsequent stabilization. In contrast, the viable cell counts of BB-12 decreased sharply by 1.13 log N/N0 within the first 4 hours of fermentation before gradually recovering to pre-inoculation levels by 16 hours. The inclusion of digested products revealed strain-specific adaptations. For *E. coli*, HM markedly enhanced growth, while CM and IF showed no significant positive or negative effects. LGG activity mirrored the ELB-0 trend under CM conditions but with amplified growth promotion. IF induced a less pronounced yet significant increase in LGG activity, whereas HM had no detectable impact. Notably, BB-12 viability was severely compromised in the presence of CM or IF, becoming undetectable, but HM supported stable survival and a 1.31 log N/N0 increase (*p* < 0.05).

**Table. 1.** Log N/N0 values from single-strain and mixed-species fermentation of *E. coli*, LGG, and BB-12, in which *N* represented the cfu/mL values after incubation and N0 represented the initial cfu/mL values before incubation. ELB-E denotes *E. coli* viable cell count in an *E. coli*-dominated mixed-species fermentation. Different capital letters represent significant differences (*p* < 0.05) between samples with different fermentation times in the same fermentation pattern.

Types of digestive products	Type of strain	Time						
		0	4	8	12	16	20	24
**0**	E	0.00^B^	0.30^A^	0.39^A^	0.36^A^	0.36^A^	0.35^A^	0.33^A^
	L	0.00^BC^	0.16^AB^	0.26^A^	0.13^AB^	−0.19^C^	−0.19^C^	−0.99^D^
	B	0.00^A^	−2.80^C^	−2.72^C^	−1.97^B^	−2.65^C^	−2.81^C^	−3.19^C^
	ELB-E	0.00^C^	0.20^BC^	0.52^AB^	0.74^A^	0.82^A^	0.73^A^	0.70^A^
	ELB-L	0.00^B^	0.00^B^	0.00^B^	0.84^A^	0.59^AB^	0.68^AB^	0.82^A^
	ELB-B	0.00^A^	−1.13^D^	−0.77^C^	−0.21^AB^	0.00^A^	−0.15^AB^	−0.29^B^
	BLE-E	0.00^A^	0.39^A^	0.16^A^	−0.04^A^	0.08^A^	−0.10^A^	0.03^A^
	BLE-L	0.00^A^	0.06^A^	0.09^A^	0.04^A^	0.23^A^	0.11^A^	0.25^A^
	BLE-B	0.00^A^	−2.55^B^	−2.28^B^	−2.48^B^	−2.38^B^	−2.36^B^	−3.21^C^
**CM**	E	0.00^B^	0.54^A^	0.59^A^	0.60^A^	0.63^A^	0.56^A^	0.06^B^
	L	0.00^A^	−0.31^B^	−0.06^A^	−0.09^A^	−0.29^B^	−0.34^B^	−0.74^C^
	B	0.00^A^	−2.59^BC^	−2.49^BC^	−2.01^BC^	−1.89^B^	−2.78^C^	−2.69^BC^
	ELB-E	0.00^A^	0.16^A^	0.06^A^	−0.04^A^	−0.17^A^	0.17^A^	−0.16^A^
	ELB-L	0.00^B^	0.05^B^	0.41^B^	0.96^A^	0.81^A^	0.87^A^	0.93^A^
	ELB-B	0.00^A^	−3.13^B^	−6.48^C^	−6.48^C^	−6.48^C^	−6.48^C^	−6.48^C^
	BLE-E	0.00^A^	−0.02^A^	−0.16^A^	−0.31^A^	−0.33^A^	−0.22^A^	0.10^A^
	BLE-L	0.00^B^	0.19^AB^	0.10^AB^	0.06^AB^	0.12^AB^	0.24^AB^	0.40^A^
	BLE-B	0.00^A^	−3.05^D^	−2.39^C^	−1.76^B^	−2.86^CD^	−3.22^D^	−3.22^D^
**IF**	E	0.00^D^	0.56^A^	0.63^A^	0.59^A^	0.51^AB^	0.34^BC^	0.20^C^
	L	0.00^A^	−0.42^AB^	−0.28^AB^	−0.71^B^	−0.42^AB^	−0.59^B^	−0.72^B^
	B	0.00^A^	−1.46^B^	−1.95^C^	−1.46^B^	−2.21^C^	−2.91^D^	−1.90^C^
	ELB-E	0.00^AB^	0.02^AB^	0.02^AB^	−0.12^B^	−0.16^B^	0.37^A^	0.04^AB^
	ELB-L	0.00^C^	0.23^BC^	0.23^BC^	0.52^AB^	0.16^BC^	0.52^AB^	0.76^A^
	ELB-B	0.00^A^	−6.48^B^	−6.48^B^	−6.48^B^	−6.48^B^	−6.48^B^	−6.48^B^
	BLE-E	0.00^A^	0.08^A^	−0.13^AB^	−0.13^AB^	−0.46^B^	−0.43^B^	0.10^A^
	BLE-L	0.00^A^	−0.04^A^	0.12^A^	−0.90^B^	−1.49^C^	−1.56^C^	0.13^A^
	BLE-B	0.00^AB^	−1.29^AB^	−4.96^A^	−4.96^C^	−4.96^B^	−4.96^C^	−4.96^A^
**HM**	E	0.00^C^	0.65^A^	0.66^A^	0.67^A^	0.56^A^	0.54^A^	0.32^B^
	L	0.00^A^	−0.67^C^	−0.18^AB^	−0.20^AB^	−0.34^B^	−0.48^BC^	−0.43^BC^
	B	0.00^A^	−1.44^BC^	−1.96^D^	−1.24^B^	−1.81^CD^	−1.86^D^	−1.96^D^
	ELB-E	0.00^BC^	0.57^AB^	0.79^A^	−0.35^C^	−0.09^BC^	0.09^BC^	0.81^A^
	ELB-L	0.00^A^	−0.06^A^	−0.11^A^	−0.19^A^	−0.22^A^	0.10^A^	−0.22^A^
	ELB-B	0.00^B^	−0.33^B^	−0.27^B^	0.83^A^	0.68^A^	0.98^A^	−0.31^B^
	BLE-E	0.00^AB^	0.04^AB^	0.02^AB^	−0.29^BC^	−0.43^C^	−0.32^BC^	0.17^A^
	BLE-L	0.00^AB^	0.04^AB^	0.15^A^	−1.34^C^	−0.27^B^	−1.63^C^	0.12^A^
	BLE-B	0.00^A^	−2.87^E^	−3.01^E^	−2.36^D^	−2.39^D^	−1.32^B^	−1.92^C^

In BB-12-dominated mixed-species fermentation (Figure 4B), *E. coli* and LGG viable cell counts remained stable without digested products (BLE-0), but BB-12 exhibited a rapid initial decline of 2.55 log N/N0 (*p* < 0.05) within 4 hours, culminating in a total reduction of 3.21 log N/N0 (*p* < 0.05) by the endpoint. CM did not alter *E. coli* survival compared to BLE-0, while IF and HM triggered an initial stabilization followed by a decline and subsequent stabilization. HM exerted the most pronounced positive effect on LGG, driving a sustained upward trend, whereas CM and IF suppressed LGG activity, with IF showing stronger inhibition. Remarkably, IF proved lethal to BB-12, while HM exerted a protective effect that maintained BB-12 viability.

### Effect of different reactor on the viable cell counts in the fermentation process

a) **Impact of reactor differences during single-strain fermentations**

**Figure 5.**
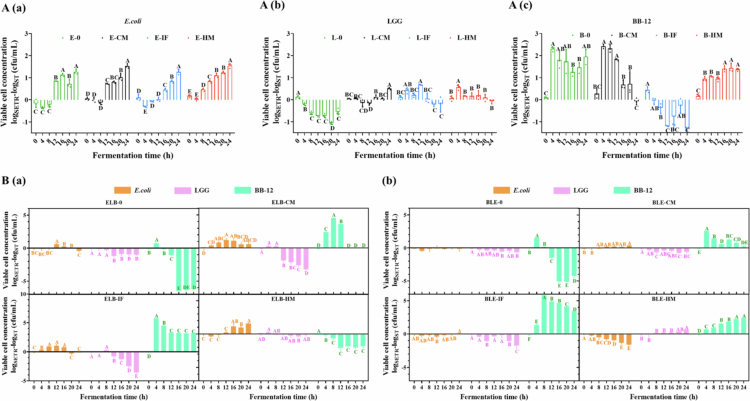
The impacts of reactor on viable cell counts during fermentation. Results are presented as log_SETR_ − log_ST_ (CFU/mL). (A) The single-strain fermentation for (a) *E. coli*, (b) LGG, and (c) BB-12. (B) (a) The mixed-species fermentation with a ratio of *E. coli*: LGG: BB-12(ELB) = 10:1:1, and (b) The mixed-species fermentation with a ratio of *E. coli*: LGG: BB-12(BLE) = 1:1:10. Different uppercase letters represent significant differences between samples at different fermentation times within the same fermentation mode (*p* < 0.05).

For *E. coli* (Figure 5A(a)), the SETR fermentation mode significantly enhanced bacterial proliferation, with overall cell viability demonstrating a marked upward trend. The presence or absence of digested products, as well as their types, exerted broadly similar effects on *E. coli* growth. In contrast, LGG viability (Figure 5A(b)) showed distinct patterns under different fermentation conditions. Compared to SETR, ST without digested products supported higher LGG survival rates. This discrepancy may stem from the gentler conditions of static fermentation (ST), where bacterial cells and metabolites settled, maintaining a dynamic equilibrium of nutrients in the medium that favored LGG viability during the initial 12 hours. Conversely, mechanical agitation in SETR promoted thorough mixing of the medium and bacterial suspension, drastically shortening the proliferation phase of LGG and triggering pronounced apoptosis after 12 hours. Notably, SETR amplified the growth-promoting effect of HM on LGG compared to other digested products. For BB-12 (Figure 5A(c)), the inhibitory effect of IF observed in prior experiments was further exacerbated under SETR conditions. In contrast, all other fermentation modes exerted a positive influence on BB-12 viability.

b) **Impact of reactor differences during mixed-species fermentations**

In *E. coli*-dominant mixed-species fermentation (Figure 5B(a)), the SETR mode further enhanced *E. coli* growth, with no discernible differences in its response to variations in digested products. For LGG, SETR exerted no significant impact on viability during the first 8 hours of fermentation (*p* < 0.05). However, beyond this period, SETR suppressed LGG proliferation in a digestion product-dependent manner. ELB-IF demonstrated the strongest inhibition, followed by ELB-CM and then ELB-0, while ELB-HM maintained LGG viability at pre-inoculation levels throughout the fermentation. Regarding BB-12, SETR effectively mitigated the cell inactivation observed in ELB-IF. Notably, however, the positive effect of ELB-HM on BB-12 viability under static (ST) conditions was not replicated in the SETR mode.

In BB-12-dominated mixed-species fermentation (Figure 5B(b)), the SETR mode exhibited marked inhibition of *E. coli* growth, particularly under HM-supplemented conditions. For LGG, SETR mirrored the viability trends observed in ST but amplified the magnitude of changes: viability declined further in BLE-0, BLE-CM, and BLE-IF, whereas BLE-HM enhanced LGG activity. Digestive product inclusion universally promoted BB-12 growth. Notably, SETR significantly ameliorated the BB-12 inactivation caused by BLE-IF. Furthermore, the negative impact of BLE-HM on BB-12 viability during the late phase of ST fermentation was substantially mitigated under SETR conditions.

### Effect of digestion products on the content of various metabolites during fermentation

Although lipid and carbohydrate species were not separately quantified during the digestion stage, the downstream fermentation profiles—including reducing sugars, amino acids, and free fatty acids—reflect digestion-mediated substrate availability and thus provide an indirect but functionally informative representation of nutrient breakdown prior to fermentation. To elucidate the metabolic mechanisms underlying the observed strain-specific viability patterns, we performed comprehensive metabolite characterization of samples from ST. Building on phenotypic observations—including pH modulation and dynamic changes in reducing sugars, free amino acids, and fatty acid content across fermentation systems—this analysis aimed to identify key biochemical pathways and metabolic intermediates associated with interactions among *E. coli*, LGG, and BB-12.

a) **Changes of pH under different fermentation protocol**

**Figure 6.**
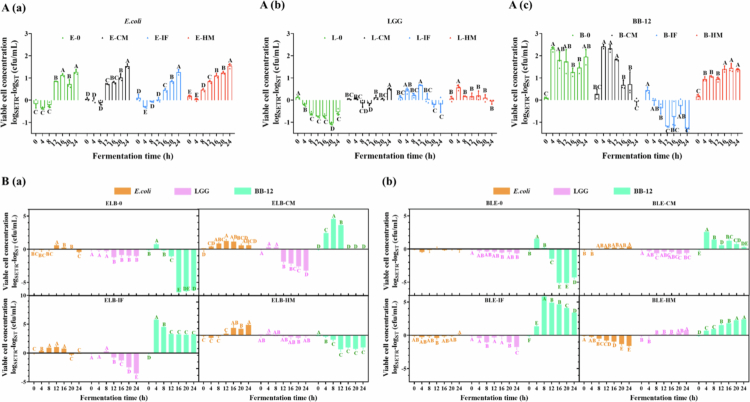
Changes in pH during fermentation with different digestion products involved. The green line represents no digested products, the blue line denotes IF-supplemented fermentation, the black line corresponds to CM-supplemented fermentation, and the red line indicates HM-supplemented fermentation.

As shown in Figure 6, the pH dynamics varied markedly across fermentation systems. In single-strain LGG fermentation, the initial pH of 5.7 decreased by 1.4, 1.6, 1.6, and 1.7 units at the endpoint for L-0, L-CM, L-IF, and L-HM, respectively. *E. coli* single-strain fermentation exhibited smaller pH reductions, with endpoint decreases of 0.6 (E-0), 0.8 (E-CM), 0.8 (E-IF), and 1.2 (E-HM). BB-12 single-strain fermentation demonstrated the mildest acidification, with final pH declines of 0.4 (B-0), 0.4 (B-CM), 0.6 (B-IF), and 0.5 (B-HM) (Figure 6A(a–c)). In *E. coli*-dominant mixed-species fermentation (ELB systems), endpoint pH reductions were 0.9 (ELB-0), 0.8 (ELB-CM), 0.9 (ELB-IF), and 1.7 (ELB-HM). Conversely, BB-12-dominant mixed fermentation (BLE systems) resulted in pH decreases of 0.7 (BLE-0), 1.5 (BLE-CM), 0.4 (BLE-IF), and 1.8 (BLE-HM). Collectively, LGG fermentation exerted the strongest acidifying effect, followed by *E. coli*, while BB-12 showed minimal impact on pH modulation. The inclusion of digested products universally enhanced environmental acidification, with HM-associated conditions consistently driving the most pronounced pH reductions across all fermentation systems (Figure 6B(a,b)).

b) **Changes of reducing sugar contents under different fermentation protocol**

**Figure 7.**
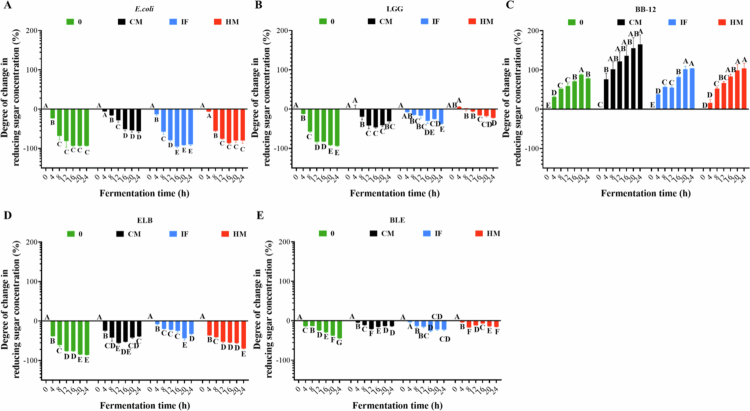
Degree of change in reducing sugars during fermentation with different digestion products involved. (A) *E. coli* single-strain fermentation. (B) LGG single-strain fermentation. (C) BB-12 single-strain fermentation. (D) *E. coli*-dominated mixed-species fermentation. (E) BB-12-dominated mixed-species fermentation. The green represents no digested products, the blue denotes IF-supplemented fermentation, the black corresponds to CM-supplemented fermentation, and the red indicates HM-supplemented fermentation. Different uppercase letters represent significant differences between samples at different fermentation times within the same fermentation mode (*p* < 0.05).

Given the variability in undigested reducing sugars across digestive products, we quantified their dynamic changes during fermentation (Figure 7A–E). Significant reductions (*p* < 0.05) were observed in all groups, with *E. coli* showing the highest sugar consumption, followed by LGG. In contrast, BB-12 promoted net sugar accumulation, particularly in HM-supplemented systems. Endpoint reductions in reducing sugar concentrations under no-digested-product conditions were 93.4% (E-0), 93.9% (L-0), −78.9% (B-0), 86.0% (ELB-0), and 44.5% (BLE-0). CM-supplemented fermentation resulted in reductions of 56.4% (E-CM), 31.2% (L-CM), −164.8% (B-CM), 39.4% (ELB-CM), and 14.0% (BLE-CM). IF-supplemented systems exhibited decreases of 89.7% (E-IF), 37.4% (L-IF), −104.0% (B-IF), 32.7% (ELB-IF), and 22.8% (BLE-IF), while HM-supplemented fermentation showed reductions of 80.5% (E-HM), 22.2% (L-HM), −103.8% (B-HM), 70.0% (ELB-HM), and 15.8% (BLE-HM). Notably, negative values indicate net production of reducing sugars, particularly observed in BB-12-associated systems. Across all conditions, *E. coli* demonstrated the highest reducing sugar consumption, followed by LGG, whereas BB-12 consistently promoted reducing sugar accumulation. The inclusion of digested products mitigated LGG-driven sugar depletion but amplified sugar accumulation in BB-12-dominated systems, suggesting strain-specific substrate utilization and metabolic cross-talk modulated by digestive residue composition.

c) **Changes of amino acid contents under different fermentation protocol**

**Figure 8.**
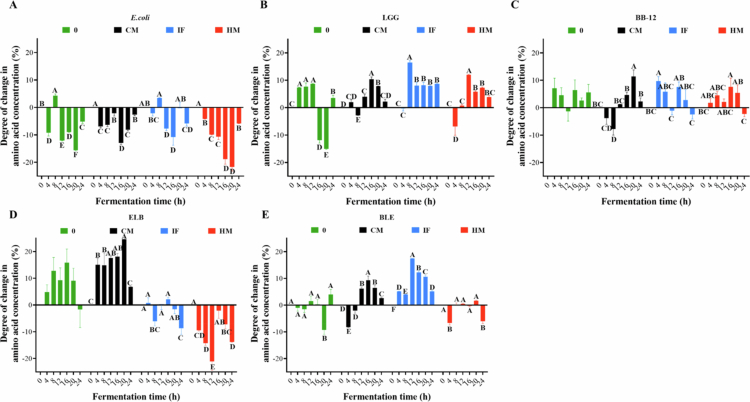
Degree of change in amino acid during fermentation with different digestion products involved. (A) *E. coli* single-strain fermentation. (B) LGG single-strain fermentation. (C) BB-12 single-strain fermentation. (D) *E. coli*-dominated mixed-species fermentation. (E) BB-12-dominated mixed-species fermentation. The green represents no digested products, the blue denotes IF-supplemented fermentation, the black corresponds to CM-supplemented fermentation, and the red indicates HM-supplemented fermentation. Different uppercase letters represent significant differences between samples at different fermentation times within the same fermentation mode (*p* < 0.05).

Figure 8(A–E) illustrates dynamic changes in amino acid content across fermentation systems. Under no-digested-product conditions, endpoint amino acid levels exhibited a net reduction of 5.1% in *E. coli* (E-0), contrasted by increases of 3.5% (L-0), 5.6% (B-0), and 4.0% (BLE-0), while ELB-0 showed a marginal decline of 1.7%. CM supplementation resulted in moderate amino acid depletion in *E. coli* (E-CM: −2.7%) and LGG (L-CM: +2.2%), with modest increases in BB-12 (B-CM: +2.2%), ELB-CM (+6.8%), and BLE-CM (+2.6%). IF-associated systems displayed pronounced strain-specific variability: *E. coli* (E-IF: −5.9%) and ELB-IF (−8.7%) showed marked depletion, whereas L-IF (+8.7%) and BLE-IF (+5.2%) demonstrated significant accumulation. HM-supplemented fermentation intensified amino acid consumption in *E. coli* (E-HM: −5.9%) and ELB-HM (−13.8%), while BLE-HM (−6.1%) shifted to net depletion, diverging from its behavior under other conditions. Collectively, *E. coli* consistently depleted amino acids, with HM further exacerbating this trend. Conversely, LGG and BB-12 generally promoted amino acid accumulation, though their impacts were modulated by digested products. Notably, CM induced a substantial amino acid increase in ELB systems, highlighting its role in enhancing nutrient availability during mixed-species fermentation. IF exerted the strongest pro-accumulation effect on BB-12-dominated systems (BLE-IF), while HM amplified cross-strain competition in *E. coli*-enriched environments (ELB-HM). These findings underscore how digestive residues reshape amino acid dynamics through strain-specific metabolic priorities and interbacterial resource competition.

d) **Changes of free fatty acid contents under different fermentation protocol**

**Figure 9.**
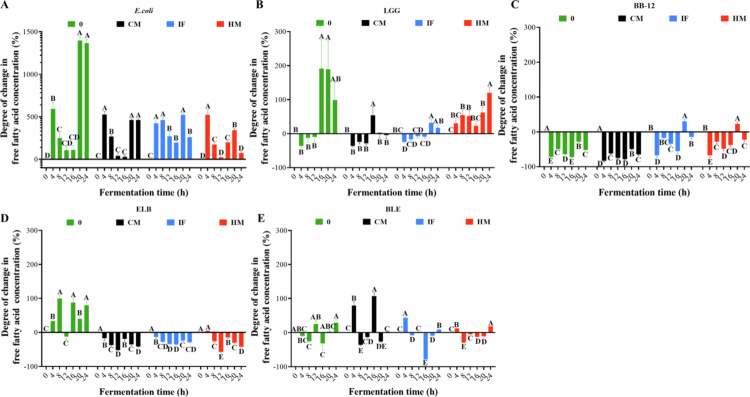
Degree of change in free fatty acid during fermentation with different digestion products involved. (A) *E. coli* single-strain fermentation. (B) LGG single-strain fermentation. (C) BB-12 single-species fermentation. (D) *E. coli*-dominated mixed-species fermentation. (E) BB-12-dominated mixed-species fermentation. The green represents no digested products, the blue denotes IF-supplemented fermentation, the black corresponds to CM-supplemented fermentation, and the red indicates HM-supplemented fermentation. Different uppercase letters represent significant differences between samples at different fermentation times within the same fermentation mode (*p* < 0.05).

Figure 9(A–E) delineates strain- and treatment-dependent variations in free fatty acid (FFA) content across fermentation systems. Under no-digested-product conditions, endpoint FFA levels increased by 1366.7% (E-0), 98.8% (L-0), 79.0% (ELB-0), and 28.5% (BLE-0), while B-0 exhibited reductions of 51.7%. CM supplementation resulted in a 462.3% FFA surge in *E. coli* (E-CM), contrasted by declines in L-CM (−4.3%), B-CM (−64.8%), ELB-CM (−41.5%), and minimal change in BLE-CM (+2.4%). IF-associated systems showed drastic FFA depletion in *E. coli* (E-IF: −252.9%) and ELB-IF (−28.8%), with modest increases in L-IF (+17.0%) and BLE-IF (+8.7%). HM-supplemented fermentation drove significant FFA accumulation in L-HM (+120.1%) and moderate increases in E-HM (+72.8%) and BLE-HM (+18.2%), while B-HM (−22.6%) and ELB-HM (−42.0%) displayed net losses. In short, *E. coli* dominated FFA production, particularly in E-0, though digested products universally attenuated this capacity. LGG exhibited digestate-dependent modulation, with HM strongly promoting FFA synthesis and static fermentation (L-0) triggering late-phase (16–24 h) FFA surges. BB-12 consistently suppressed FFA accumulation, with CM exerting the strongest inhibition (B-CM). Mixed-species fermentation mitigated FFA flux magnitude compared to single-strain systems, suggesting cross-regulatory metabolic interactions. Notably, IF and CM exerted statistically indistinguishable effects on LGG-driven FFA dynamics (*p* > 0.05), underscoring HM’s unique role in enhancing lipolytic activity. Because the three selected strains (*E. coli*, LGG, BB-12) are not major producers of classical short-chain fatty acids, SCFA quantification was not expected to provide additional mechanistic insight in this defined three-strain model.

## Discussion

This study investigated the *in vitro* gastrointestinal digestion of CM, IF, and HM, with subsequent fermentation of their digestive residues to assess strain-specific responses in *E. coli*, LGG, and BB-12. The dynamic shifts in pH (Figure 6), reducing sugars (Figure 7), amino acids (Figure 8), and FFAs (Figure 9) observed across fermentation systems serve as critical biomarkers of microbial metabolic activity and substrate prioritization.

### Higher protein degradation in HM

The hydrolysis patterns of dietary proteins during digestion critically influenced their bioavailability and subsequent microbial utilization. Notably, *α*-lactalbumin—a predominant protein in HM—exhibited minimal gastric hydrolysis but underwent extensive tryptic cleavage in the intestinal phase, yielding small peptides and free amino acids.^26^ Conversely, *β*-lactoglobulin demonstrated partial gastric hydrolysis followed by trypsin-mediated intestinal breakdown,^27^ while casein formed gastric gel matrices that delayed gastric emptying and sustained gradual intestinal proteolysis, generating casein-derived peptides.^28^ HM’s unique protein profile, including structurally stable lactoferrin and immunoglobulins with preserved secondary/tertiary configurations, conferred resistance to rapid proteolytic degradation.^29^^,^^30^ These structural distinctions were reflected in CM electrophoretic banding patterns, where HM and IF casein/whey hydrolysis profiles diverged markedly from residual protein signatures.

### HM digestion residues have a smaller particle size

Particle size dynamics further differentiated digestive outcomes: casein’s propensity to form large gastric coagulates increased residual particle size, whereas HM’s elevated lactose and oligosaccharide content enhanced protein solubility, reducing aggregate formation.^31^ These physicochemical properties likely modulated microbial access to nutrients—casein-derived peptides and larger particles may have acted as sustained-release substrates, while HM’s soluble components facilitated rapid assimilation. Such mechanistic distinctions align with our observations of strain-specific metabolic preferences (e.g., *E. coli*’s heightened amino acid consumption in HM systems) and competitive dynamics in mixed cultures.

### HM bioactive drive strain-specific microbial dynamics: synergistic suppression of *E. coli* and niche optimization for probiotics

BB-12 displayed poor persistence in nutrient-limited conditions, consistent with its known reliance on exogenous carbohydrates and peptides for metabolic activity. In contrast, *E. coli*-maintained viability under the same conditions, reflecting its metabolic versatility and ability to exploit minimal substrates. These baseline differences highlight the distinct ecological strategies of the three strains and provide a framework for interpreting how digestion residues modulated their responses.

Across fermentation systems, HM digestion products consistently supported LGG and BB-12 while limiting *E. coli* growth. While this pattern aligns with the bioactive-rich nature of HM reported in previous literature, our study did not quantify specific components such as HMOs, lactoferrin, lysozyme, or immunoglobulins. Therefore, the selective effects observed here cannot be attributed to any individual molecule or molecular pathway. Instead, they likely arise from the overall biochemical environment generated after HM digestion, including its unique balance of carbohydrates, small peptides, organic acids, and partially hydrolyzed proteins.^32^^,^^33^ This nutrient and pH landscape appears to create competitive conditions that favor LGG and BB-12 over *E. coli* in our simplified fermentation model.

Within this context, the enhanced persistence of LGG and BB-12 under HM-conditioned conditions is best interpreted as a functional outcome of substrate composition, rather than definitive evidence of HMO-driven prebiotic activity, bioactive peptide function, or selective antimicrobial action. Likewise, the suppression of *E. coli* under HM conditions reflects resource competition and acidification dynamics rather than confirmed actions of lactoferrin or lysozyme. These findings therefore represent strain-level metabolic tendencies, not mechanistic demonstrations of HM bioactivity.

In mixed-species systems, the suppression of *E. coli* by LGG and BB-12 was evident, yet this should be understood as an interaction within a constrained three-strain model. While similar competitive relationships between probiotics and Enterobacteriaceae are observed *in vivo*, real infant gut ecosystems involve diverse and interdependent taxa that can substantially modify cross-feeding, niche occupation, and metabolic fluxes. Thus, although our findings parallel known patterns in breastfed infants, they cannot be generalized to whole-community behavior without caution.^34^

Future studies incorporating targeted quantification of specific HM components or multi-omics analyzes in more complex microbial consortia will be necessary to determine which biochemical features of HM digestion products are responsible for shaping microbial interactions. For now, the results highlight that digestion residue composition—rather than any single molecular factor—governs strain-specific survival and competition in early-stage fermentation environments.

### Reactor configurations modulate strain-specific adaptations and microbial interactions

The differential effects of reactor configurations (SETR vs. ST) on microbial viability and inter-strain competition highlight how physical mixing conditions shape fermentation behavior within simplified gut models (Figure 5). In single-strain fermentations, *E. coli* displayed consistently higher viability under SETR operation, whereas LGG maintained greater persistence in static environments.^35^ These patterns likely reflect fundamental physiological differences—*E. coli* tolerates dynamically mixed environments well, while LGG, which is adapted to more quiescent niches, exhibited reduced stability under continuous agitation.^36^ Importantly, these interpretations remain phenomenological: although factors such as oxygen exposure, shear sensitivity, or nutrient dispersion may contribute, the present study did not directly quantify these variables and therefore cannot delineate their mechanistic roles.^37^

Across fermentation systems, BB-12 demonstrated comparatively higher stability under SETR operation than under ST, producing an estimated ~23% increase in overall viable cell output. This difference reflects an empirical outcome rather than a defined metabolic mechanism; SETR’s cyclic compression produces more homogeneous mixing than ST, which may help maintain more uniform access to nutrients or reduce local metabolite accumulation, but these possibilities remain speculative. What can be concluded from our data is that BB-12 responds more favorably to the dynamic environment created by SETR than to the static conditions of ST.

In mixed-species fermentations, reactor-dependent differences further altered community trajectories. SETR amplified *E. coli* dominance in ELB systems, while the survival of BB-12 in ELB-IF was partially restored relative to ST. These observations suggest that mixing conditions modulate the balance between strains, potentially by influencing resource distribution or contact-dependent interactions; however, the specific drivers of these shifts were not measured and therefore cannot be definitively attributed to metabolic or physical mechanisms. Similarly, the reduced persistence of BB-12 in HM-supplemented SETR fermentations compared with static conditions indicates that the advantages conferred by HM digestion products are context-dependent and sensitive to reactor configuration.

The selective suppression of *E. coli* observed in HM-supplemented BLE systems under both ST and SETR conditions supports the broader pattern that HM-conditioned substrates favor probiotic survival relative to *E. coli* across multiple contexts. While prior literature attributes such effects to HM-associated immune proteins and complex carbohydrates, the current study did not quantify these factors; thus, the observed inhibition should be interpreted conservatively as an emergent property of HM digestion residues rather than evidence of specific bioactive mechanisms.

Overall, our findings illustrate that SETR and ST environments yield distinct microbial outcomes even when inoculated with identical strains and substrates. Rather than functioning as mechanistic analogs of gastrointestinal motility, these reactors impose different physical regimes that can alter nutrient accessibility, spatial organization, and interspecies interactions in ways that directly influence fermentation outcomes. Because only three strains were used, these results should be viewed as insights into strain-specific adaptation within simplified communities, not as direct proxies for the behavior of more complex infant gut consortia. Future work incorporating multi-omics measurements and additional taxa will be necessary to clarify which reactor-dependent effects arise from physical mixing conditions and which reflect underlying metabolic interactions.

### Dietary substrate composition modulates microbial fitness through pH dynamics, and nutrient partitioning in infant gut ecosystems

pH fluctuations, driven by organic acid production (e.g., lactate, acetate), reflect net acidification, while reducing sugar depletion highlights strain-specific carbon source utilization. Amino acid profiles reveal proteolytic activity and nitrogen competition, and FFA accumulation or depletion signals lipid metabolism and energy storage strategies. These collective changes delineate strain-specific metabolic niches and illustrate how digestive residues reshape resource partitioning. In the absence of digestive products, restricted metabolic activity led to diminished acid production, minimal pH variation, and gradual acidification. CM or IF supplementation, rich in lactose and proteins, provided *E. coli* with auxiliary carbon sources, stimulating metabolic activity and accelerating pH reduction.^38^ Conversely, IF’s protein and lipid content initially buffered acid production in LGG, resulting in attenuated pH shifts.^39^ HM’s composition—lactose, oligosaccharides, and lipids—significantly amplified metabolic activity, particularly in *E. coli*, driving prolific acidogenesis and the most pronounced pH decline.^40^ This aligns with LGG and BB-12’s capacity to generate SCFAs, such as acetate, propionate, and lactate, which acidify the gut environment. In contrast, *E. coli*’s metabolic functions and membrane integrity are compromised under low-pH conditions,^41^ explaining its suppressed growth in HM systems. The stability of LGG and BB-12 viability in HM-supplemented systems likely stems from their evolutionary adaptation to acidic niches, where SCFA production reinforces their competitive edge. HM’s oligosaccharides may further enhance this resilience by serving as bifidogenic substrates, while its antimicrobial components (e.g., lactoferrin) selectively target *E. coli* without disrupting probiotic activity.^8^^,^^42^ These patterns suggest that digestive residues modulate amino-acid dynamics through strain-dependent substrate utilization and cross-strain interactions.

The differential utilization of reducing sugars among strains highlights distinct metabolic strategies shaped by dietary substrates. LGG efficiently metabolizes lactose-derived glucose and galactose into lactic acid,^43^ but unlike BB-12, it cannot utilize HMOs, leading to higher reducing sugar consumption in IF- and CM-supplemented systems. In contrast, BB-12 metabolizes HM- and IF-derived oligosaccharides, generating intermediate reducing sugars that transiently elevate their concentrations.^10^ HM’s HMOs provide BB-12 with a preferential carbon source, reducing cross-strain competition for lactose and stabilizing reducing sugar levels. Conversely, IF’s lack of HMOs forces all strains to compete for lactose, accelerating reducing sugar depletion.^38^ CM’s simplified composition (lactose, casein, whey) further intensifies lactose-driven reducing sugar consumption by LGG and *E. coli*, resulting in sharp declines. These observations underscore how dietary matrices dictate colonic sugar dynamics through strain-specific substrate preferences.

Amino acid profiles revealed niche-specific nitrogen acquisition strategies. LGG’s amino acid demand correlates with its growth phase, where increased viability drives proteolytic activity to meet biosynthetic needs. HM-supplemented systems maintained elevated amino acid levels (*p* < 0.05), likely due to LGG’s reliance on exogenous essential amino acids and HM’s rich peptide content.^44^ BB-12, despite its robust amino acid utilization capacity,^45^ showed minimal net depletion in HM systems, as its efficient uptake balanced HM’s sustained nutrient supply. *E. coli* preferentially degraded dietary proteins over amino acids, with HM’s complex proteome supporting greater amino acid catabolism than IF or CM.^46^ However, this did not translate to enhanced viability, suggesting metabolic trade-offs between nitrogen assimilation and energy production via TCA cycle intermediates.^47^

Free fatty acid (FFA) dynamics were influenced by pH-mediated lipase activity and substrate complexity. *E. coli*’s weaker acidification in HM systems created a permissive pH environment for lipase function, yet HM’s diverse substrates (oligosaccharides, hydrolyzed proteins) diverted metabolic resources away from FFA production.^48^ Conversely, IF’s protein-lipid matrix initially buffered acid stress, prolonging lipase activity in LGG. BB-12’s consistent FFA suppression across conditions aligns with its limited lipolytic capacity and prioritization of carbohydrate metabolism. Although this study did not quantify specific SCFAs or bile acids, the analysis of total FFA content offers insight into microbial lipid metabolism under various fermentation conditions. FFAs themselves play important roles in modulating immune responses and epithelial integrity. The strain-dependent FFA dynamics observed particularly the elevated production by *E. coli* in static conditions and the suppressive effect of BB-12, suggest distinct metabolic strategies with potential relevance to host-microbe signaling. Future studies incorporating targeted metabolomics for SCFAs and bile acid derivatives could further elucidate the immune-modulatory potential of these fermentation systems.

### Study limitations

Several limitations should be acknowledged when interpreting these findings. First, the three-strain model used here captures only a narrow subset of the taxonomic and functional diversity present in the infant gut microbiome. While it enables controlled assessment of strain-specific metabolic behavior, community-level processes—such as cross-feeding, competitive exclusion, and ecological succession—cannot be fully resolved without more complex microbial consortia. Second, our metabolite profiling focused on primary nutrient classes (reducing sugars, amino acids, and free fatty acids). Although these data provide insight into broad metabolic strategies, they do not reveal the contributions of secondary metabolites—including SCFAs, bile acids, vitamins, and quorum-sensing molecules—that often drive microbe–microbe and microbe–host interactions. Third, as an *in vitro* fermentation system, the model does not incorporate host-derived factors such as immune regulation, epithelial transport, mucus adhesion, or neuroendocrine signaling, all of which substantially influence microbial fitness in vivo. Finally, the 24-hour fermentation window was selected to approximate infant colonic transit and capture early metabolic responses; however, longer-term fermentations may be required to study stabilization dynamics, resilience, and metabolite succession. These limitations highlight the need for future studies combining multi-strain communities, expanded metabolomic profiling, and in vivo validation to contextualize the ecological and nutritional relevance of the present observations.

## Conclusion

This study systematically examined how gastrointestinal digestion products from human milk, infant formula, and a control milk matrix modulate the growth and metabolic activity of *Lactobacillus rhamnosus* GG, *Bifidobacterium animalis* subsp. *lactis* BB-12, and *Escherichia coli* within both static and peristalsis-mimicking fermentation environments. Across all experiments, each strain exhibited distinct metabolic priorities—*E. coli* preferentially consumed amino acids and produced substantial free fatty acids, LGG displayed strong utilization of reducing sugars with notable acidification activity, and BB-12 contributed to reducing sugar accumulation while consistently suppressing fatty acid release. Digestion products further shaped these behaviors: HM- and IF-derived residues supported higher probiotic persistence and altered pH, sugar, amino acid, and lipid turnover patterns, whereas CM exerted more variable effects. The comparison between the static reactor and SETR highlighted the influence of mechanical forces, with SETR enhancing nutrient dispersion and promoting the metabolic output of some strains while reducing the viability of others. These findings demonstrate how substrate composition and reactor conditions jointly regulate strain-specific ecological strategies even in a simplified system. Importantly, the present study does not identify molecular mechanisms but instead provides foundational evidence of how digestion-derived nutrient landscapes shape microbial responses at the single-species and mixed-species levels. These findings from a simplified model system require validation using more complex microbial communities and ultimately *in vivo* studies to determine their broader physiological relevance.

## Supplementary Material

Supplementary material.docx Supplementary material.docx

## Data Availability

The raw data obtained from this experiment can be accessed online at https://data.mendeley.com/datasets/3jg3z44fnj/1.
