# Supplementary material for: Using in vitro gastrointestinal (GI) tract digestive products to investigate fermentation dynamics of Lactobacillus rhamnosus GG, Bifidobacterium animalis subsp. lactis BB-12, and Escherichia coli
Source: Gut Microbes Rep. 2026 Jan 26;3(1):2620183. doi: 10.1080/29933935.2026.2620183 (PMC12938877; doi:10.1080/29933935.2026.2620183)
Supplement: Supplementary material.docx [file KGMR_A_2620183_SM6031.docx]

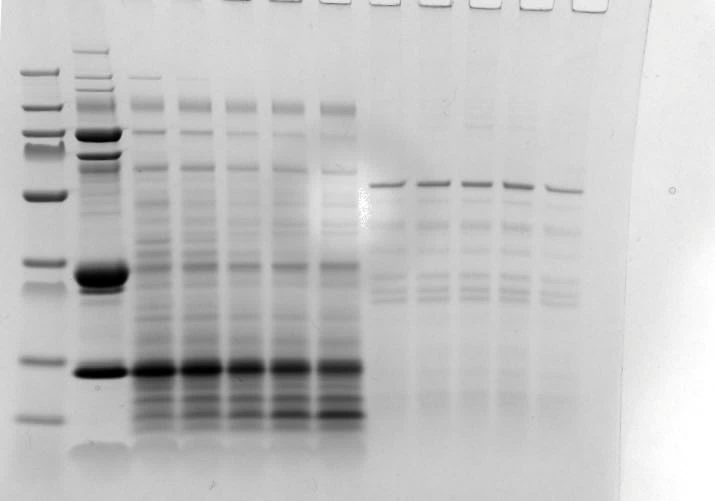


Figure S1. Original image of SDS-PAGE analysis of protein profiles during gastrointestinal digestion of HM. The first lane is the molecular weight marker, with bands corresponding to 250, 130, 100, 70, 55, 35, 25, 15, and 10 kDa from top to bottom. Lanes 2 to 11 represent: undigested HM, gastric digestion at 0 min, 30 min, 60 min, 90 min, and 120 min, followed by intestinal digestion at 0 min, 30 min, 60 min, 90 min, and 120 min, respectively.
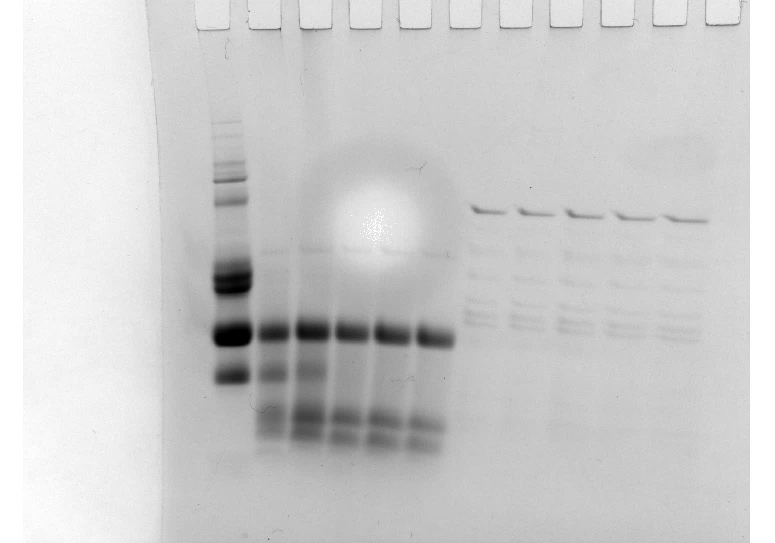


Figure S2. Original image of SDS-PAGE analysis of protein profiles during gastrointestinal digestion of IF. Lanes 1 to 11 represent: undigested IF, gastric digestion at 0 min, 30 min, 60 min, 90 min, and 120 min, followed by intestinal digestion at 0 min, 30 min, 60 min, 90 min, and 120 min, respectively.


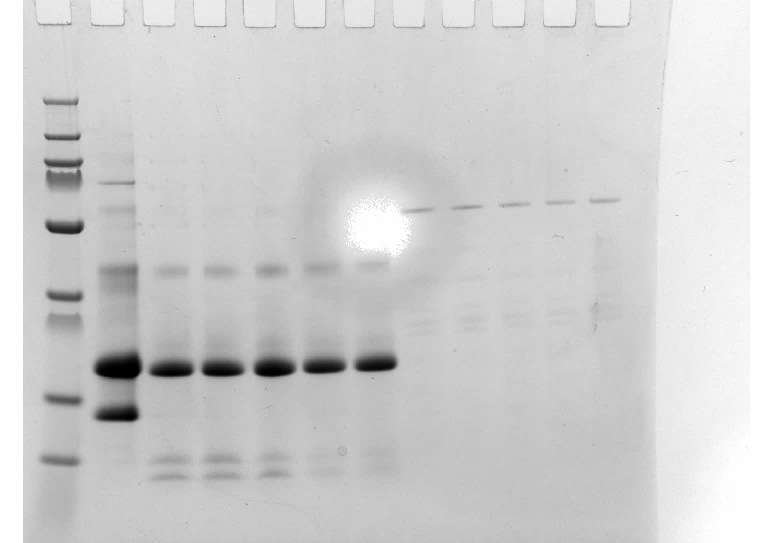


Figure S3. Original image of SDS-PAGE analysis of protein profiles during gastrointestinal digestion of CM. The first lane is the molecular weight marker, with bands corresponding to 250, 130, 100, 70, 55, 35, 25, 15, and 10 kDa from top to bottom. Lanes 2 to 11 represent: undigested CM, gastric digestion at 0 min, 30 min, 60 min, 90 min, and 120 min, followed by intestinal digestion at 0 min, 30 min, 60 min, 90 min, and 120 min, respectively.


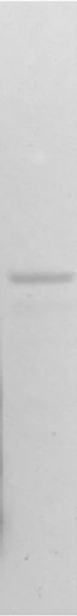


Figure S4. Original SDS-PAGE analysis of simulated gastric fluid.


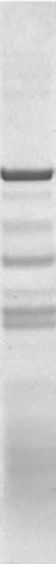


Figure S5. Original SDS-PAGE analysis of simulated intestinal fluid.

Table S1. Nutritional composition of the infant formula (IF) used in this study.

|  | Unit | Per 100kJ | Per 100g | Per 100mL |
| --- | --- | --- | --- | --- |
| Energy | kJ | 100 | 2079 | 274 |
| Protein | g | 0.50 | 10.4 | 1.4 |
| Fat | g | 1.31 | 27.3 | 3.6 |
| Linoleic acid | g | 0.15 | 3.15 | 0.42 |
| α- Linoleic acid | mg | 25 | 519 | 69 |
| Carbohydrates | g | 2.38 | 49.4 | 6.5 |
| Vitamin A | micrograms of retinol equivalents | 17.8 | 370 | 29 |
| Vitamin D | μg | 0.37 | 7.7 | 1.02 |
| Vitamin E | mg α-tocopherol equivalent | 0.38 | 7.9 | 1.04 |
| Vitamin E_1_ | μg | 1.30 | 27 | 3.56 |
| Vitamin B_1_ | μg | 18.1 | 377 | 50 |
| Vitamin B_2_ | μg | 26 | 542 | 72 |
| Vitamin B_6_ | μg | 14.5 | 302 | 40 |
| Vitamin B_12_ | μg | 0.032 | 0.66 | 0.087 |
| Niacin | μg | 131 | 2720 | 359 |
| Folic Acid | μg | 4.16 | 86.4 | 11.4 |
| Pantothenic Acid | μg | 109 | 2274 | 300 |
| Vitamin C | mg | 3.08 | 64 | 8.4 |
| Biotin | μg | 0.46 | 9.6 | 1.27 |
| Sodium | mg | 6.5 | 134.1 | 17.7 |
| Potassium | mg | 21 | 441 | 58 |
| Copper | μg | 14.5 | 302 | 40 |
| Magnesium | mg | 1.74 | 36.1 | 4.77 |
| Iron | mg | 0.19 | 4.00 | 0.53 |
| Zinc | mg | 0.16 | 3.42 | 0.45 |
| Manganese | μg | 2.79 | 58 | 7.7 |
| Calcium | mg | 17.1 | 355 | 47 |
| Phosphorus | mg | 12.2 | 254 | 34 |
| Iodine | μg | 3.50 | 72.8 | 9.6 |
| Chloride | mg | 15.2 | 316 | 42 |
| Selenium | μg | 0.63 | 13.0 | 1.72 |
| Choline | mg | 3.25 | 67.5 | 8.9 |
| Inositol | mg | 1.44 | 30 | 3.96 |
| Taurine | mg | 1.73 | 36 | 4.75 |
| L - Carnitine | mg | 0.42 | 8.8 | 1.16 |
| Docosahexaenoic Acid (DHA) |  | 0.33 | 0.33 | 0.33 |
| Arachidonic Acid (ARA) |  | 0.33 | 0.33 | 0.33 |
| Galacto-Oligosaccharides (GOS) | g | 0.26 | 5.49 | 0.72 |
| Fructo - Oligosaccharides (FOS) | g | 0.029 | 0.61 | 0.081 |
| Nucleotides | mg | 1.04 | 21.6 | 2.85 |

Table S2. Normality testing and statistical procedures applied for all experimental datasets.

| **Dataset / Variable** | **Group/Condition** | **Shapiro–Wilk p-value** | **Normality Met (Yes/No)** | **Statistical Test Used** | **Multiple Comparison Correction** |
| --- | --- | --- | --- | --- | --- |
| Log CFU | ST-E-0 | 0.000109 | No | Friedman test | Benjamini–Hochberg FDR |
| Log CFU | ST-E-CM | 0.000916 | No | Friedman test | Benjamini–Hochberg FDR |
| Log CFU | ST-E-IF | 0.016449 | No | Friedman test | Benjamini–Hochberg FDR |
| Log CFU | ST-E-HM | 0.000641 | No | Friedman test | Benjamini–Hochberg FDR |
| Log CFU | ST-L-0 | 0.00094 | No | Friedman test | Benjamini–Hochberg FDR |
| Log CFU | ST-L-CM | 0.00192 | No | Friedman test | Benjamini–Hochberg FDR |
| Log CFU | ST-L-IF | 0.50273 | Yes | Repeated-measures ANOVA | Bonferroni |
| Log CFU | ST-L-HM | 0.16026 | Yes | Repeated-measures ANOVA | Bonferroni |
| Log CFU | ST-B-0 | 1.90 × 10⁻⁵ | No | Friedman test | Benjamini–Hochberg FDR |
| Log CFU | ST-B-CM | 0.002192 | No | Friedman test | Benjamini–Hochberg FDR |
| Log CFU | ST-B-IF | 0.518733 | Yes | Repeated-measures ANOVA | Bonferroni |
| Log CFU | ST-B-HM | 0.077875 | Yes | Repeated-measures ANOVA | Bonferroni |
| Log CFU | ST-ELB-0-E | 0.002923 | No | Friedman test | Benjamini–Hochberg FDR |
| Log CFU | ST-ELB-0-L | 0.005126 | No | Friedman test | Benjamini–Hochberg FDR |
| Log CFU | ST-ELB-0-B | 0.003758 | No | Friedman test | Benjamini–Hochberg FDR |
| Log CFU | ST-ELB-CM-E | 0.215288 | Yes | Repeated-measures ANOVA | Bonferroni |
| Log CFU | ST-ELB-CM-L | 0.014506 | No | Friedman test | Benjamini–Hochberg FDR |
| Log CFU | ST-ELB-CM-B | 2.80 × 10⁻⁶ | No | Friedman test | Benjamini–Hochberg FDR |
| Log CFU | ST-ELB-IF-E | 0.05544 | Yes | Repeated-measures ANOVA | Bonferroni |
| Log CFU | ST-ELB-IF-L | 0.58845 | Yes | Repeated-measures ANOVA | Bonferroni |
| Log CFU | ST-ELB-IF-B | 4.39 × 10⁻⁸ | No | Friedman test | Benjamini–Hochberg FDR |
| Log CFU | ST-ELB-HM-E | 0.59237 | Yes | Repeated-measures ANOVA | Bonferroni |
| Log CFU | ST-ELB-HM-L | 0.03555 | No | Friedman test | Benjamini–Hochberg FDR |
| Log CFU | ST-ELB-HM-B | 0.00610 | No | Friedman test | Benjamini–Hochberg FDR |
| Log CFU | ST-BLE-0-E | 0.17285 | Yes | Repeated-measures ANOVA | Bonferroni |
| Log CFU | ST-BLE-0-L | 0.54665 | Yes | Repeated-measures ANOVA | Bonferroni |
| Log CFU | ST-BLE-0-B | 3.64 × 10⁻⁵ | No | Friedman test | Benjamini–Hochberg FDR |
| Log CFU | ST-BLE-CM-E | 0.17836 | Yes | Repeated-measures ANOVA | Bonferroni |
| Log CFU | ST-BLE-CM-L | 0.23253 | Yes | Repeated-measures ANOVA | Bonferroni |
| Log CFU | ST-BLE-CM-B | 0.000875 | No | Friedman test | Benjamini–Hochberg FDR |
| Log CFU | ST-BLE-IF-E | 0.13609 | Yes | Repeated-measures ANOVA | Bonferroni |
| Log CFU | ST-BLE-IF-L | 0.005159 | No | Friedman test | Benjamini–Hochberg FDR |
| Log CFU | ST-BLE-IF-B | 2.75 × 10⁻⁶ | No | Friedman test | Benjamini–Hochberg FDR |
| Log CFU | ST-BLE-HM-E | 0.09220 | Yes | Repeated-measures ANOVA | Bonferroni |
| Log CFU | ST-BLE-HM-L | 0.21469 | Yes | Repeated-measures ANOVA | Bonferroni |
| Log CFU | ST-BLE-HM-B | 0.009910 | No | Friedman test | Benjamini–Hochberg FDR |
| Reducing sugar | E-0 | 0.00202 | No | Friedman test | Benjamini–Hochberg FDR |
| Reducing sugar | E-CM | 0.03729 | No | Friedman test | Benjamini–Hochberg FDR |
| Reducing sugar | E-IF | 0.00578 | No | Friedman test | Benjamini–Hochberg FDR |
| Reducing sugar | E-HM | 0.00199 | No | Friedman test | Benjamini–Hochberg FDR |
| Reducing sugar | L-0 | 0.00243 | No | Friedman test | Benjamini–Hochberg FDR |
| Reducing sugar | L-CM | 0.02351 | No | Friedman test | Benjamini–Hochberg FDR |
| Reducing sugar | L-IF | 0.00108 | No | Friedman test | Benjamini–Hochberg FDR |
| Reducing sugar | L-HM | 0.03354 | No | Friedman test | Benjamini–Hochberg FDR |
| Reducing sugar | B-0 | 0.12532 | Yes | Repeated-measures ANOVA | Bonferroni |
| Reducing sugar | B-CM | 0.18891 | Yes | Repeated-measures ANOVA | Bonferroni |
| Reducing sugar | B-IF | 0.19136 | Yes | Repeated-measures ANOVA | Bonferroni |
| Reducing sugar | B-HM | 0.22527 | Yes | Repeated-measures ANOVA | Bonferroni |
| Reducing sugar | ELB-0 | 0.00231 | No | Friedman test | Benjamini–Hochberg FDR |
| Reducing sugar | ELB-CM | 0.01258 | No | Friedman test | Benjamini–Hochberg FDR |
| Reducing sugar | ELB-IF | 0.00344 | No | Friedman test | Benjamini–Hochberg FDR |
| Reducing sugar | ELB-HM | 0.01672 | No | Friedman test | Benjamini–Hochberg FDR |
| Reducing sugar | ELB-0 | 0.00231 | No | Friedman test | Benjamini–Hochberg FDR |
| Reducing sugar | ELB-CM | 0.01258 | No | Friedman test | Benjamini–Hochberg FDR |
| Reducing sugar | ELB-IF | 0.00344 | No | Friedman test | Benjamini–Hochberg FDR |
| Reducing sugar | ELB-HM | 0.01672 | No | Friedman test | Benjamini–Hochberg FDR |
| Amino acids | E-0 | 0.3507 | Yes | Repeated-measures ANOVA | Bonferroni |
| Amino acids | E-CM | 0.3391 | Yes | Repeated-measures ANOVA | Bonferroni |
| Amino acids | E-IF | 0.6408 | Yes | Repeated-measures ANOVA | Bonferroni |
| Amino acids | E-HM | 0.2407 | Yes | Repeated-measures ANOVA | Bonferroni |
| Amino acids | L-0 | 0.00648 | No | Friedman test | Benjamini–Hochberg FDR |
| Amino acids | L-CM | 0.4883 | Yes | Repeated-measures ANOVA | Bonferroni |
| Amino acids | L-IF | 0.0514 | Yes | Repeated-measures ANOVA | Bonferroni |
| Amino acids | L-HM | 0.7645 | Yes | Repeated-measures ANOVA | Bonferroni |
| Amino acids | B-0 | 0.9075 | Yes | Repeated-measures ANOVA | Bonferroni |
| Amino acids | B-CM | 0.9161 | Yes | Repeated-measures ANOVA | Bonferroni |
| Amino acids | B-IF | 0.1902 | Yes | Repeated-measures ANOVA | Bonferroni |
| Amino acids | B-HM | 0.9690 | Yes | Repeated-measures ANOVA | Bonferroni |
| Amino acids | ELB-0 | 0.9866 | Yes | Repeated-measures ANOVA | Bonferroni |
| Amino acids | ELB-CM | 0.2728 | Yes | Repeated-measures ANOVA | Bonferroni |
| Amino acids | ELB-IF | 0.0975 | Yes | Repeated-measures ANOVA | Bonferroni |
| Amino acids | ELB-HM | 0.5264 | Yes | Repeated-measures ANOVA | Bonferroni |
| Amino acids | BLE-0 | 0.0669 | Yes | Repeated-measures ANOVA | Bonferroni |
| Amino acids | BLE-CM | 0.3991 | Yes | Repeated-measures ANOVA | Bonferroni |
| Amino acids | BLE-IF | 0.1865 | Yes | Repeated-measures ANOVA | Bonferroni |
| Amino acids | BLE-HM | 0.00315 | No | Friedman test | Benjamini–Hochberg FDR |
| Free fatty acids | E-0 | 0.00365 | No | Friedman test | Benjamini–Hochberg FDR |
| Free fatty acids | E-CM | 0.01061 | No | Friedman test | Benjamini–Hochberg FDR |
| Free fatty acids | E-IF | 0.31125 | Yes | Repeated-measures ANOVA | Bonferroni |
| Free fatty acids | E-HM | 0.10628 | Yes | Repeated-measures ANOVA | Bonferroni |
| Free fatty acids | L-0 | 0.0110 | No | Friedman test | Benjamini–Hochberg FDR |
| Free fatty acids | L-CM | 0.0463 | No | Friedman test | Benjamini–Hochberg FDR |
| Free fatty acids | L-IF | 0.2994 | Yes | Repeated-measures ANOVA | Bonferroni |
| Free fatty acids | L-HM | 0.3730 | Yes | Repeated-measures ANOVA | Bonferroni |
| Free fatty acids | B-0 | 0.02296 | No | Friedman test | Benjamini–Hochberg FDR |
| Free fatty acids | B-CM | 0.00320 | No | Friedman test | Benjamini–Hochberg FDR |
| Free fatty acids | B-IF | 0.82916 | Yes | Repeated-measures ANOVA | Bonferroni |
| Free fatty acids | B-HM | 0.87199 | Yes | Repeated-measures ANOVA | Bonferroni |
| Free fatty acids | ELB-0 | 0.26087 | Yes | Repeated-measures ANOVA | Bonferroni |
| Free fatty acids | ELB-CM | 0.31686 | Yes | Repeated-measures ANOVA | Bonferroni |
| Free fatty acids | ELB-IF | 0.03073 | No | Friedman test | Benjamini–Hochberg FDR |
| Free fatty acids | ELB-HM | 0.40719 | Yes | Repeated-measures ANOVA | Bonferroni |
| Free fatty acids | BLE-0 | 0.98082 | Yes | Repeated-measures ANOVA | Bonferroni |
| Free fatty acids | BLE-CM | 0.00996 | No | Friedman test | Benjamini–Hochberg FDR |
| Free fatty acids | BLE-IF | 0.00820 | No | Friedman test | Benjamini–Hochberg FDR |
| Free fatty acids | BLE-HM | 0.51003 | Yes | Repeated-measures ANOVA | Bonferroni |
